# Enhancing Vasculogenesis in Dental Pulp Development: DPSCs-ECs Communication via FN1-ITGA5 Signaling

**DOI:** 10.1007/s12015-024-10695-6

**Published:** 2024-02-28

**Authors:** Tiankai Di, Chao Feng, Lulu Wang, Jinlong Xu, Yang Du, Baixiang Cheng, Yujiang Chen, Lian Wu

**Affiliations:** 1 State Key Laboratory of Oral & Maxillofacial Reconstruction and Regeneration, National Clinical Research Center for Oral Diseases, Shaanxi Clinical Research Center for Oral Diseases, Department of Pediatric Dentistry, School of Stomatology, The Fourth Military Medical University, Xi’an, Shaanxi 710032 People’s Republic of China; 2 Department of Stomatology, No.969 Hospital, Joint Logistics Support Force of the Chinese People’s Liberation Army, Hohhot, Inner Mongolia 010000 People’s Republic of China; 3 Center for Computational Biology, Institute of Military Cognition and Brain Sciences, Academy of Military Medical Sciences, Beijing, 100850 People’s Republic of China; 4 Department of Clinical Laboratory, No.969 Hospital, Joint Logistics Support Force of the Chinese People’s Liberation Army, Hohhot, Inner Mongolia 010000 People’s Republic of China; 5 Key Laboratory of Shaanxi Province for Craniofacial Precision Medicine Research, Clinical Research Center of Shaanxi Province for Dental and Maxillofacial Diseases, Department of General Dentistry, College of Stomatology, Xi’an Jiaotong University, Xi’an, Shaanxi 710032 People’s Republic of China; 6 Department of Neurobiology and Institute of Neurosciences, School of Basic Medicine, Fourth Military Medical University, Xi’an, Shaanxi 710032 People’s Republic of China

**Keywords:** Single-cell RNA sequencing, Dental pulp stem cells, Vascular endothelial cell, Intercellular communication, Vascularization

## Abstract

**Background:**

Dental pulp regeneration therapy is a challenge to achieve early vascularization during treatment. Studying the regulatory mechanisms of vascular formation during human dental pulp development may provide insights for related therapies. In this study, we utilized single-cell sequencing analysis to compare the gene expression of dental pulp stem cells (DPSCs) and vascular endothelial cells (ECs) from developing and mature dental pulps.

**Method:**

Immunohistochemistry, Western blot, and real-time polymerase chain reaction (RT-PCR) were used to detect fibronectin 1 (FN1) expression and molecules, such as PI3K/AKT. Cell proliferation assay, scratch assay, tube formation assay and were used to investigate the effects of DPSCs on the vasculogenetic capability of ECs. Additionally, animal experiments involving mice were conducted.

**Result:**

The results revealed that DPSCs exist around dental pulp vasculature. FN1 expression was significantly higher in DPSCs from young permanent pulps than mature pulps, promoting HUVEC proliferation, migration, and tube formation via ITGA5 and the downstream PI3K/AKT signaling pathway.

**Conclusion:**

Our data indicate that intercellular communication between DPSCs and ECs mediated by FN1-ITGA5 signaling is crucial for vascularizationduring dental pulp development, laying an experimental foundation for future clinical studies.

**Graphical Abstract:**

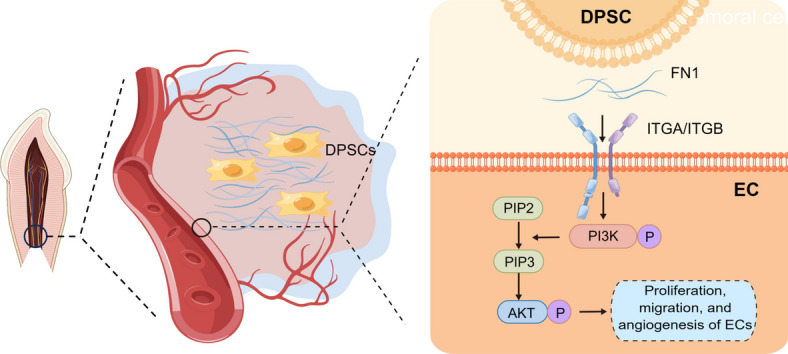

**Supplementary Information:**

The online version contains supplementary material available at 10.1007/s12015-024-10695-6.

## Introduction

Caries, dental trauma, and abnormal tooth development are common causes of dental pulp diseases, which can lead to severe consequences, such as loss of pulp vitality and interrupted tooth development [1]. In contrast to traditional techniques, such as root canal therapy, pulp regeneration attempts to regenerate dental pulp tissues and bring hope to patients with dental pulp disease [2]. Many studies have successfully applied autologous stem cell homing, stem cell transplantation, exosome therapy, or scaffold-based strategies for pulp regeneration [3–7]. Promoting vasculogenesis of the dental pulp is pivotal to achieving pulp regeneration and restoring its physiological functions. However, rapid vascularization of engineered tissues remains challenging in current pulp regeneration.

The dental pulp is a highly vascularized tissue in a non-expandable environment surrounded by hard dentin walls, with the root canal as the only passageway. The vascular architecture of the human dental pulp develops during human development, serving as a nutrition supply and waste removal and playing an important role in pulp inflammation reactions and subsequent regeneration [8]. Exploring the pulp vascular formation during development may provide a new approach to regenerative therapies. Previous studies have described the histopathology and gene expression of the pulp development in temporal and spatial dimensions [8–10]. Current researches attempt to explore the mechanisms of pulp vascular formation during tooth development and apply developmental mechanisms to clinical practice in pulp tissue engineering [11–13]. However, due to lack of a comprehensive and deep understanding of the specific regulatory mechanisms during pulp development, it is difficult to effectively guide pulp tissue regeneration through developmental approaches so far.

Regulation of the pulp vascular formation is complex and precise. Specifically, intercellular communication between endothelial cells (ECs) and various surrounding cells may be a key factor in inducing vascular formation [14]. Therefore, exploring the regulatory processes between ECs and other cells during vascular development can provide insights into effective pathways for pulp regeneration. Studies have demonstrated that dental pulp stem cells (DPSCs) are key contributors to pulp homeostasis and injury repair, with great potential in pulp regenerative therapies. Multiple studies have focused on pulp regeneration using DPSCs and their derived exosomes, cell aggregates, and endogenous stem cell homing. Researchers have observed the spatial coexistence between pulp stem cells and vasculature, suggesting possible direct or indirect regulatory relationships [15]. For example, studies showed that stem cells from human exfoliated deciduous teeth (SHEDs) can exhibit specific perivascular cell characteristics, and SHEDs and human umbilical vein endothelial cells (HUVECs) express angiogenic factors and corresponding receptors, respectively, thereby mediating interactions between perivascular cells and ECs [16]. Additionally, TGF-β1-treated DPSCs can regulate vascularizationin three-dimensionally co-cultured HUVECs and DPSCs via vascular endothelial growth factor(VEGF)-Ang-Tie2 signal transduction [17]. However, in the past our tools were insufficient to map the intercellular communication landscape of ECs during pulp development to identify key signaling pathways. Single-cell RNA sequencing (scRNA-seq) technology can analyze the contributions of different cell subtypes and intercellular communication to physiological functions and pathogenic mechanisms at the single-cell level, providing a viable alternative to traditional methods.

This study utilized single-cell sequencing data analysis to compare the DPSC and EC gene expressions in developing and mature dental pulps, as well as differences in intercellular communication between them, to explore and demonstrate the regulatory role of DPSC-EC intercellular communication on vasculogenesis during pulp development, laying an experimental foundation for future clinical applications in tissue engineering regeneration.

## Materials and Methods

### scRNA-seq data collection and processing

In this study, 40,266 cells, including 26,459 from mature permanent tooth (Adult Group, *n* = 5) and 13,807 from Young permanent teeth(Young Group, *n* = 2), were investigated. Detailed information is provided in Supplementary Table S1. The single-cell RNA expression matrices were downloaded from the Gene Expression Omnibus (GEO) public database (GEO: GSE146123). All dataset samples were prepared as outlined by the 10 × Genomics Single Cell 3′ Reagent Kit user guide. The sequencing reads were processed using the CellRanger (10 × Genomics) analysis pipeline.

### Quality control and analysis

The “Seurat” R package (V4.1.1) was applied for quality control procedures and downstream bioinformatic analyses. Doublets were removed from each sample using the R package DoubletFinder with a loose assumption (3% doublet rate). Low-quality cells satisfying any of the following criteria were filtered: Unique Molecular Markers(UMIs) < 500 or UMIs > 5000 and the proportion of mitochondrial gene counts (> 15%). Following these quality control procedures, a series of preprocessing procedures were performed for downstream analysis. Specifically, a global scaling normalization method, "Log-Normalize," that normalized the feature expression for each cell, was performed to preprocess the total expression and multiply it by a scaling factor (10,000 by default). The result was log-transformed using the “NormalizeData()” function in Seurat. Afterward, the normalized expression profiles of all samples were merged using the “merge()” function in R v3.6.3. The different datasets were integrated using harmony (v0.1.0), designed to remove the influence of technical effects between datasets and cells and identify shared cell states across different datasets. After the integration, the Seurat package was used to analyze scRNA-seq data, including identification of highly variable genes, unsupervised graph-based clustering, differentially expressed genes (DEGs), and dimension reduction using principal component analysis and Uniform Manifold Approximation and Projection.

### Identification of signature genes for cell clusters

The DEGs in each subcluster were identified using the “FindAllMarkers” function in Seurat. The significance levels of these signature genes were determined using the Wilcoxon rank-sum test and Bonferroni correction. The signature genes of each cluster were determined using the following criteria: (1) expressed in more than 20% of the cells within either or both two groups; (2) |log_2_FC |> 0.5; and (3) Wilcoxon rank-sum test adjusted P-value < 0.01.

### Pathway enrichment analysis

An analysis of Gene Ontology (GO) and Kyoto Encyclopedia of Genes and Genomes (KEGG) was performed using the “clusterProfiler” R package v4.0.2 to investigate the potential functions of different cell types [18]. Pathways with P_adj-values < 0.05 were considered significantly enriched. The mean gene expression of each cell type was included as input data using the gene set variation analysis (GSVA) package v1.34.0 for the GSVA and the pathway enrichment analysis [19].

### Cell–cell communication analysis with Cellchat

Cell–cell communication was analyzed and visualized using CellChat v1.1.0 (github.com/sqjin/CellChat) [20]. The cell type labels were derived from the Harmony integration results using all scRNA-seq data sources. The default values were used for each step parameterization.

### Tooth sample

The premolars of 9- to 10-year-old children were extracted due to orthodontic treatment. The healthy mature premolars of 18-year-old patients were extracted due to orthodontic treatment. Tooth mobility was checked clinically. Moreover, the history of drug allergy, systemic disease, and family genetic disease were excluded. After tooth extraction, the teeth were categorized into Adult and Young pulp groups. Finally, the number of teeth at each stage of the study was analyzed histologically as follows: A: 10, Y: 10. Additionally, three randomly selected teeth at each stage were used for subsequent cell experiments. All tooth samples were obtained from the Stomatology and Maxillofacial Surgery of the Stomatological Hospital of the Fourth Military Medical University of the Chinese People's Liberation Army. This study was approved by the Ethics Committee of the Stomatological Hospital of the Fourth Military Medical University of the Chinese People's Liberation Army. Furthermore, the guardian of each participant signed an informed consent form.

### Histological examination

HE staining was performed using the detection protocol of the HE staining kit (Solarbio, G1120). Immunofluorescence staining was performed on the HE-stained sections to quantify and compare each protein expression in tooth pulp samples. Primary antibodies (anti-CD31 antibody (Abcam, ab28364), anti-FN1 antibody (Abcam, ab2413), anti-Integrin alpha-5 antibody (Abcam, ab275977), fibronectin receptors were formed by binding ITGA5 and ITGB1), and anti-CD105 (Abcam, ab252345)) was incubated at 1: 200 for 2 h, followed by a secondary antibody (Goat Anti-Rabbit IgG H&L, Alexa Fluor® 555, (Abcam, USA, at 1/200 dilution)) for 1 h at room temperature. The slides were sealed in a mounting medium containing 4',6-diamidino-2-phenylindole(DAPI, Vector Laboratories, H1200, dilution to 10μg/ml) for further image acquisition. Images were captured using a microscope (Leica, M205FA, Wetzlar, Germany), and mean optical densities of contrast tissue sections were quantified using ImageJ (https://imagej-nih-gov.laneproxy.stanford.edu/ij/).

### Cell culture

Following collecting tooth samples, the teeth were sterilized and split, and the pulp was extracted. After washing with phosphate buffer saline(PBS), the pulp tissue was cut into no more than 1 mm^3^ pieces. Then, they were digested with collagenase type I (Sigma, USA) at 37 °C for 60 min, centrifuged, and re-suspended in α-MEM (Gibco, USA) medium. They were seeded in a six-well culture plate and incubated at 37 ℃ and 5% CO_2_. When primary cells reached 80% confluence, trypsinization (Sigma, USA) was used for passage. After counting and diluting the first-generation cells to a cell density of 10–15 cells/mL in α-MEM medium, they were pipetted into a 96-well plate, and 150 µL of cell suspension was added to each well. After overnight incubation at a constant temperature, the wells containing only a single cell were labeled, 100 µL of the culture medium was added, and incubation was continued. When the cell clones occupied half the well bottom, the digested monoclonal cells were transferred to a six-well plate for expansion. HUVECs were purchased from Lonza (Basel, Switzerland) and cultured in an endothelial cell medium (ECM, ScienceCell, USA). All cells were incubated under 5% CO_2_ at 37 ℃.

### Osteogenic and adipogenic induction

Each group’s fourth-generation cells were trypsinized and seeded in a six-well plate at a density of 3 × 10^3^ cells/well, and 2 mL of α-MEM medium containing 5% fetal bovine serum (FBS) was added to each well and placed at a constant temperature in a cell incubator. After 24 h, each well was replaced with osteogenic induction medium (α-MEM medium with 5% FBS, dexamethasone 10 mol/L, ascorbic acid Vc 50 µg/mL, and β-glycerophosphate sodium 10 mmol/L; Sigma-Aldrich) and adipogenic induction medium (α-MEM medium with 5% FBS, dexamethasone 0.25 µmol/L, indomethacin 100 mmol/L, IBMY 0.5 mmol/L, and insulin 10 mg/L; Sigma-Aldrich) to continue culturing. The medium was changed every three days for three to four weeks during induction. Each group was stained with alizarin red and oil red O (Sigma-Aldrich). When staining, the cell induction medium was aspirated, washed three times with PBS, and filtered with 4% paraformaldehyde (Beyotime, China) for 20 min. Then, the medium was discarded and washed three times with PBS. After adding alizarin red or oil and drying red O, cells were incubated at 37 ℃ for 2 h. After discarding the dye, the sample was rinsed three times with PBS and photographed under an inverted microscope.

### HUVECs and DPSCs co-culture

HUVECs and DPSCs were collected and suspended in a conditioned HUVECs and α-MEM medium, respectively. HUVECs were trypsinized, and the cell suspension was prepared in ECM. HUVECs (4 × 10^5^) were seeded onto the upper surface of the Transwell insert membrane. DPSCs were trypsinized, and the cell suspension was prepared in a growth medium. DPSCs (4 × 10^5^) were seeded into the lower chamber of the transwell plate. After 24 h of seeding, the upper and lower chambers medium was replaced with a fresh growth medium, and cells were lysed for experiments.

### ELISA

ELISA was used to compare the fibronectin 1 (FN1) expression in DPSCs. All procedures followed the manufacturer's instructions (FineTest, EH0134). Briefly, triplicate supernatants were collected at passage four from routinely cultured DPSCs. The FN1 concentration was quantified in the supernatant using an ELISA kit. The kit’s standard was used as a reference. The experiment was repeated three times to verify the results.

### RNA interference

DPSCs (1 × 10^5^) were inoculated into a 24-well plate containing an appropriate amount of complete medium (464.50 µL) to achieve transfection density of 30%–50%. Afterward, 1.25 µL of 20 µM siRNA (Thermo Fisher) stock solution was diluted with 30 µL 1X riboFECsTTM CP Buffer (Ribobio) and 3 µL riboFECsTTM CP Reagent (Ribobio) to prepare a mixture. The mixture was incubated at room temperature for 15 min. The mixture was added to the cell culture medium, and the plate was incubated in a CO_2_ incubator at 37 ℃. The mRNA expression was detected using qPCR after 24 h, and the protein expression was detected using Western blot after 72 h.

### Recombinant FN1 and PI3K inhibitors

Recombinant human FN1 (Solarbio, P00167) was reconstituted in PBS containing 0.1% BSA to a working concentration of 50 ng/mL. The PI3K inhibitor LY294002 (Abcam, ab120243) was used at a concentration of 20 μM [21].

### Real-time polymerase chain reaction (RT-PCR)

Trizol (Invitrogen, USA) was used to extract the total RNA of DDPSCs and DPSCs in each group of the fourth generation, and cDNA was synthesized using PrimeScript™ RT-PCR kit (Takara, China). Furthermore, reverse transcription was conducted according to the instructions. The cDNA template obtained by reverse transcription was added to the detection system according to the RT-PCR reagent operating instructions, with cDNA as a template and GAPDH as an internal reference. The ABI7500 fluorescence RT-qPCR system (Applied Biosystems, Germany) was used to detect FN1. The reaction conditions for OPG expression were as follows: pre-denaturation at 95 ℃ for 30 s, one cycle; 95 ℃ (denaturation) for 5 s; and 60 ℃ (annealing and extension) for 30 s, 40 cycles. The experiment was repeated three times. The primer sequences are listed in Table 1.

**Table 1:** Sequences of primers for each molecular gene amplification

Genes	Primers
FN1 GAPDH	F: CCGCCGAATGTAGGACAAGA R: GACAGAGTTGCCCACGGTAA F: GCACCGTCAAGGCTGAGAAC R: TGGTGAAGACGCCAGTGGA

### Western blot detection

The DPSCs and HUVECs of each group were treated with protein lysis buffer (10 mM Tris–HCL, 1 mM EDTA, 1% sodium dodecyl sulfate, 1% Nonidet P-40, 1:100 proteinase inhibitor cocktail, 50 mM b-glycerophosphate, and 50 mM sodium fluoride). After completely lysing, the resulting cytoplasm was treated using an ultrasonic cell disrupter (4 W) for 5 s on ice. Following sonication, the samples were ice-bathed for 5 min, centrifuged at 25,000 rpm at 4 ℃ for 10 min, and the supernatant was collected to obtain total protein. The proteins were extracted using a Nuclear Extract Kit (Sangon Biotech, China). The protein concentration was measured using a Pierce® BCA Protein Assay Kit (Beyotime, China). The loading amount was weighted according to the determined protein sample concentration to ensure the same amount of protein in each well, and electrophoresis was conducted using 10% SDS-PAGE. After electrophoresis, the protein was transferred to the PVDF membrane (Millipore). Additionally, 1:1000 diluted primary antibodies (anti-FN1 antibody (Abcam, ab2413,), anti-p-PI3K (Abcam, ab278545), anti-PI3K (Abcam, ab154598), Anti-AKT (Abcam, ab179463), and Anti-p-AKT (Abcam, ab81283)) were added and incubated at 1/500 dilution at 4 ℃ overnight. The membranes were washed with TBST and incubated with a secondary antibody (Goat Anti-Rabbit IgG H&L, Alexa Fluor® 555, (Abcam, ab150078, at 1/2000 dilution)) for 1 h at room temperature. Immunoreactive bands were detected using enhanced chemiluminescence (ECL) according to the manufacturer's instructions. The experiment was repeated three times. The average gray value of the scanned bands was determined using ImageJ software.

### Cell viability assay

The cell viability was determined using the CCK-8 assay. Briefly, 5 × 10^3^ cells were seeded into 96-well culture plates and allowed to adhere overnight. Then, the cells were changed to a fresh medium containing various Andro concentrations (5, 10, 25, and 50 μM) dissolved in DMSO (final concentration, less than 0.1%). After incubating for 48 h, CCK-8 was added, and the absorbance was measured at 450 nm using EnSpire® Multimode Plate Reade (Perkin Elmer, USA). The cell viability in vehicle control groups was considered 100%. Each assay was conducted at least in triplicate.

### In vitro migration assay

Scratch assay (wound healing assay) was performed to detect cell migration ability. The HUVECs were grown to full confluence in six-well plates. The cell monolayers were wounded with a sterile 200 μL pipette tip and then washed with PBS after 6 h-starvation. The cells were changed to fresh medium (5% FBS) containing indicated doses of Andro, with or without 50 ng/mL VEGF. After 12 h, the medium was replaced with PBS, the wound gap was observed, cells were photographed using a Leica DM14000B microscope fitted with a digital camera, and the distance between the wound gaps was measured.

### Tube formation assay

Tube Formation Assay was used to evaluate the effects of DPSCs on in vitro vasculogenesis. HUVECs were cultured in an ECM (ScienCell, USA). For the tube formation assay, growth factor reduced Matrigel (Corning, USA) was pipetted into each well of a pre-chilled six-well plate and polymerized at 37 °C for 30 min. Then, HUVECs were seeded onto the Matrigel at 2 × 10^4^ cells/well density. The cells were treated with conditioned medium collected from DPSCs cultures or cultured in ECM alone as a control. After 6 h of incubation, tube formation of HUVECs was observed under an inverted microscope. The degree of tube formation was quantified by measuring the total tube length in five randomly selected fields per well using ImageJ software.

### Animal experiments

Six- to eight-week-old male C57BL/6J mice were provided by the Fourth Military Medical University Animal Center (Xi'an, China). Animal experiments in this study were approved by the Animal Care and Experiment Committee of the Fourth Military Medical University (approval number: IACUC-20240005). Forty mice were randomly divided into four groups to establish the model for tooth development in vivo study. Briefly, The experimental animals were divided into four groups: Adult control group(postnatal day 28, root growth completed), Young control group(postnatal day 14, root growth uncompleted, periodontal membrane injection of mandibular first molar since postnatal day 8, 50 uL saline buffer, administered three times weekly for 1 week), Young group with siRNA-FN1(injection method same as previous group, 10 nmol siRNA-FN1 in 50 uL saline buffer), Young group with siRNA-ITGA5(injection method same as previous group, 10 nmol siRNA- ITGA5 in 50 uL saline buffer),. To evaluate pulp development, mice were sacrificed, and the mandible were subjected to histological analyses. All animals were given free access to sterilized food and water and were habituated for a week before the experiments. All procedures were carried out in strict accordance with the recommendations established by Animal Care and Ethics Committee of the Fourth Military Medical University as well as the guidelines by U.S. National Institutes of Health Guide for Care and Use of Laboratory Animals. The protocol was approved by the Animal Care and Ethics Committee of the Fourth Military Medical University.

## Statistical Analysis

All statistical analyses of single-cell sequencing data were implemented using R statistical programming language (V.3.62). The continuous variables denoted as mean ± SD were compared using the Wilcoxon test. The categorical data presented as percentage (%) were compared using χ^2^-test or Fisher’s exact test. A two-sided P < 0.05 was considered statistically significant. Computer statistical analysis software, SPSS 22.0, was used to analyze the obtained data. Differences between data groups were analyzed using one-way ANOVA. The turkey test was used for pairwise comparisons between groups. A P < 0.05 indicated that the difference was statistically significant.

## Results

### Landscapes of young and adult dental pulps

We extracted seven samples, five from mature permanent teeth and two from young permanent teeth, from the GSM database to explore the spatial and temporal characteristics of human pulp gene expression at different developmental stages (Extended Data Fig. 1A). We classified all pulp tissue cells into eight types using SingleR and previous studies (Extended Data Fig. 1B). COL1A1 and LUM were used to define fibroblasts, DMP1 to define odontoblasts, and THY1/CD90 to define MSCs (defined as DPSCs). CD3D was used to define lymphocytes, and CD68 and VWF were used to label mononuclear phagocytes (MPs) and ECs. PLP1 and MKI67 were used to label glial and proliferating cells, respectively.

### Transcriptional differences of ECs from young and adult human dental pulp

**Fig. 1:**
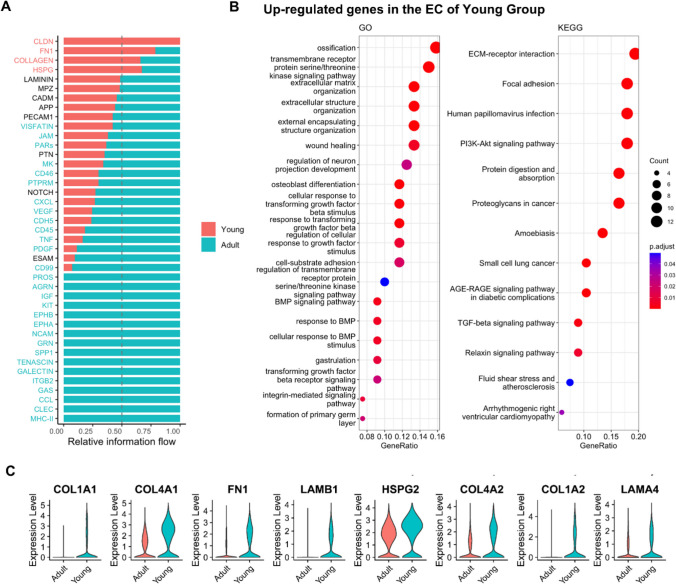
Transcriptional differences of ECs from young and adult human dental pulp. **A**: The transcriptomic analysis results of mature and young dental pulp tissues. **B**: GO and KEGG analysis results of DEGs in ECs from mature and young dental pulp tissues. **C**: DEGs involved in extracellular matrix receptor interactions in ECs

Transcriptomic differences can reflect changes in tissue biological functions. We sought differences between mature and young dental pulp tissues from overall dental pulp and EC perspectives. Initially, we compared overall transcriptome analysis results of mature and young dental pulp tissues. The results presented that genes closely related to biological functions, such as cell–matrix connections, cell adhesion, and migration processes, including CLDN, FN1, and COLLAGEN, were highly expressed in young dental pulp tissues (Fig. 1A). Additionally, we analyzed the DEGS of ECs using GO and KEGG pathway databases. The results exhibited that highly expressed genes in ECs from young dental pulp tissues were mainly enriched in extracellular matrix receptor interaction, cell adhesion, wound healing, and TGF-β stimulated cell response (Fig. 1B). These results are also consistent with the biological functions of young dental pulp tissues that are still growing and developing. Finally, we disclosed the enrichment of DEGs involved in extracellular matrix receptor interactions between the two EC groups (Fig. 1C), hoping to identify specific mechanisms regulating the biological functions of ECs in permanent dental pulp through further research (Fig. 1).

### Effects of co-culture with DPSCs on the biological functions of HUVECs

Previous studies have demonstrated that DPSCs are frequently spatially distributed in dental pulp tissue around blood vessels, and DPSCs can regulate vascular functions in various ways [22]. Therefore, in this part of the experiment, we studied the effects of DPSCs from mature permanent teeth (Adult-DPSCs, A-DPSCs) and young permanent teeth (Young-DPSCs, Y-DPSCs) on ECs' biological functions.

**Fig. 2:**
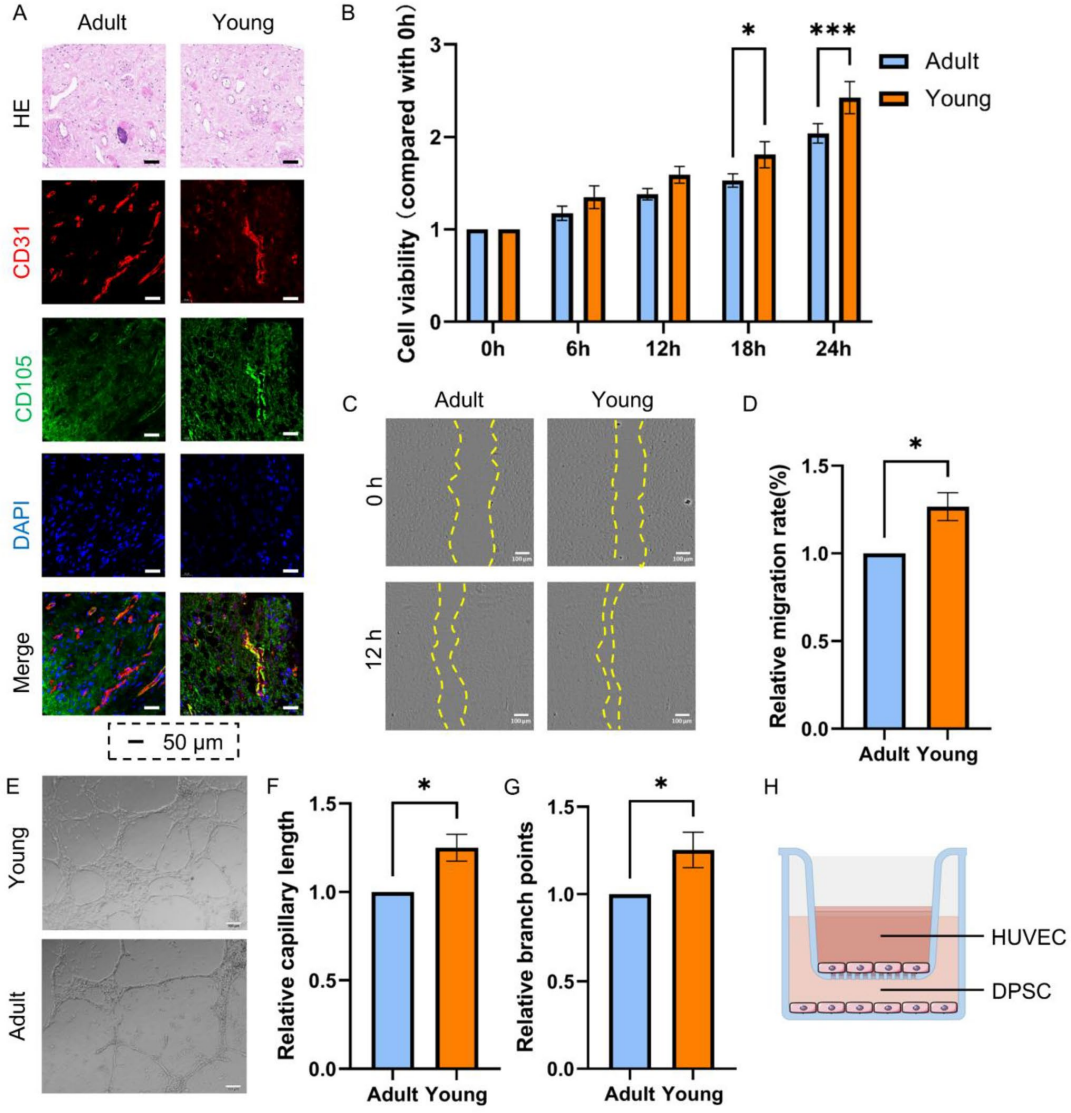
Effects of co-culture with DPSCs on the biological functions of HUVECs. **A**: HE staining and CD31/CD105 immunofluorescence staining of mature and young dental pulp tissues. **B**: Proliferative ability of HUVECs in A-DPSCs and Y-DPSCs co-cultured groups was determined using CCK-8 assay. **C**–**D**: Cell migration of HUVECs co-cultured with DPSCs in a scratch assay. **E**–**H**: The tube formation assay was performed by counting branch points and capillary length. I: A-DPSCs and Y-DPSCs co-cultured with HUVECs using transwell inserts. (*P < 0.05, **P < 0.01, ***P < 0.001)

The dental pulp tissue immunofluorescence results showed the presence of DPSCs (CD105 +) around blood vessels (CD31 +), with higher CD105 expression around blood vessels in young dental pulp tissues (Fig. 2A). This also demonstrates the potential regulatory relationship between DPSCs and ECs, which may be the source of biological functional differences between ECs from different groups.

Therefore, we extracted DPSCs primary cells from young and mature dental pulps and conducted an in vitro passaging culture. DPSC-EC co-culture experiments were conducted using transwell inserts or conditioned medium methods (Fig. 2H). CCK8 assays of HUVECs were conducted and analyzed. The results presented that HUVECs co-cultured with Y-DPSCs had stronger proliferative capacity (Fig. 2B). Scratch test results displayed that HUVECs co-cultured with Y-DPSCs healed faster than the A-DPSCs co-cultured group (Fig. 2C/D). The HUVEC tube formation results disclosed that the tube formation ability of the Y-DPSCs co-cultured group was stronger (Fig. 2E–G). Based on this section’s results, we can speculate that Y-DPSCs may affect the biological functions of HUVECs via intercellular regulatory effects, thereby promoting vasculogenesis (Fig. 2).

### Intercellular Communication between DPSCs and ECs in Dental Pulp

**Fig. 3:**
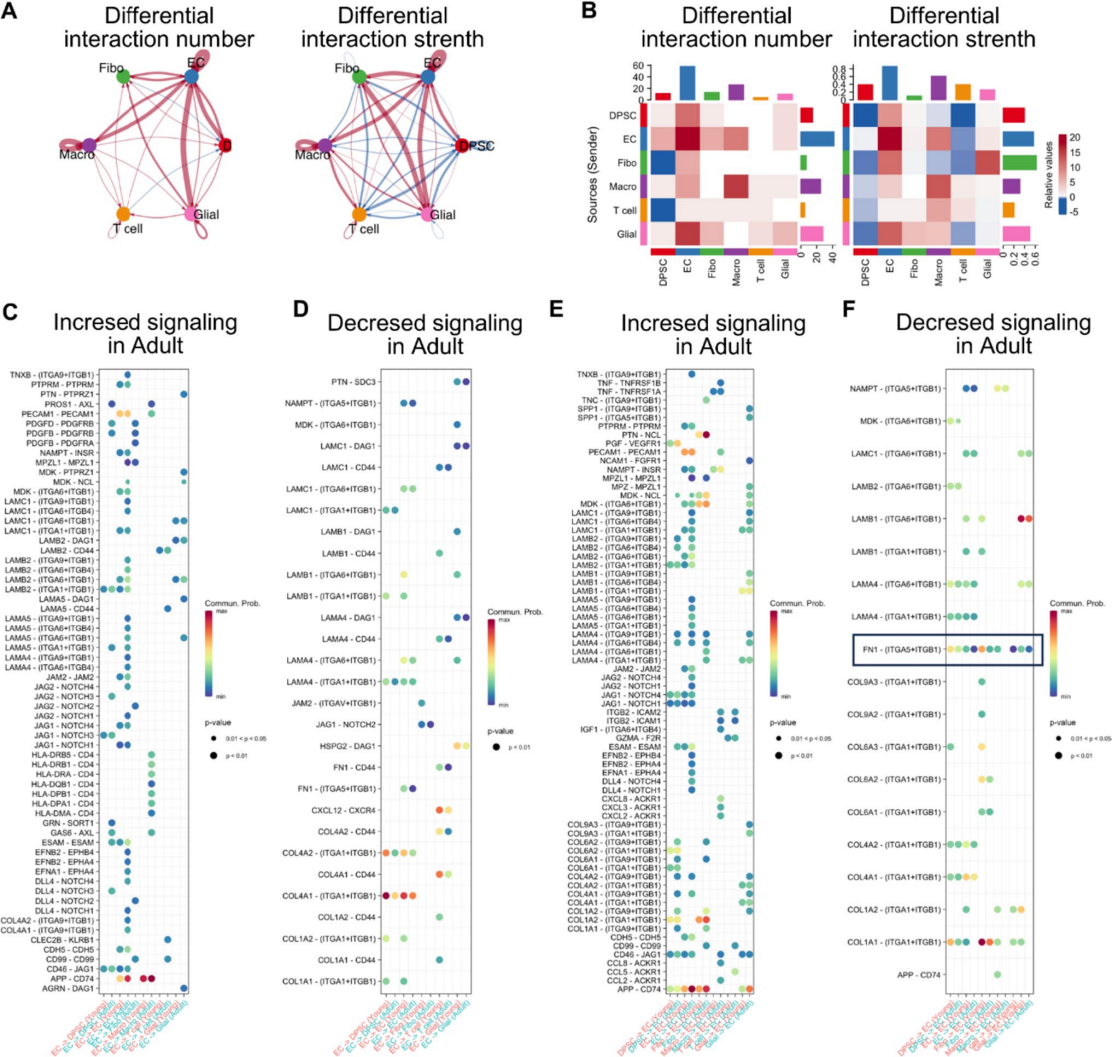
Analysis of intercellular communication of ECs in young permanent and mature permanent dental pulp tissues. **A**/**B**: Interaction strength and numbers of interactions in intercellular communication in mature and young permanent dental pulp tissues. **C**: More active intercellular communication pathways (regulatory effects of ECs on other cell types) in mature permanent dental pulp tissue. **D**: More active intercellular communication pathways (regulatory effects of ECs on other cell types) in young permanent dental pulp tissue. **E**: More active intercellular communication pathways (regulatory effects of other cell types on ECs) in mature permanent dental pulp tissue. F: More active intercellular communication pathways (regulatory effects of other cell types on ECs) in young permanent dental pulp tissue

The interaction between DPSCs and ECs involves intercellular communication [23]. We utilized single-cell sequencing data's cell–cell communication analysis results to comprehensively explore intercellular communication between DPSCs and ECs in dental pulp tissue. Initially, we comprehensively described intercellular communication between different cell types in dental pulp tissue (Fig. 3A/B). Additionally, we provided intercellular communication pathways with activity differences between young and mature permanent dental pulps, including regulatory effects of ECs on other cell types (Fig. 3C/D) and regulatory effects of other cell types on ECs (Fig. 3E/F). This section’s results discovered that the FN1-ITGA5 signaling pathway in DPSC-EC intercellular communication was more active in young permanent dental pulp (Fig. 3F). FN1 is an important extracellular matrix component with wide-ranging effects on recipient cell biology functions. Therefore, we further explored the specific roles of the FN1-ITGA5 signaling pathway in DPSC-EC intercellular communication (Fig. 3).

### Spatial expression of FN1/ITGA5 in dental pulp tissue

**Fig. 4:**
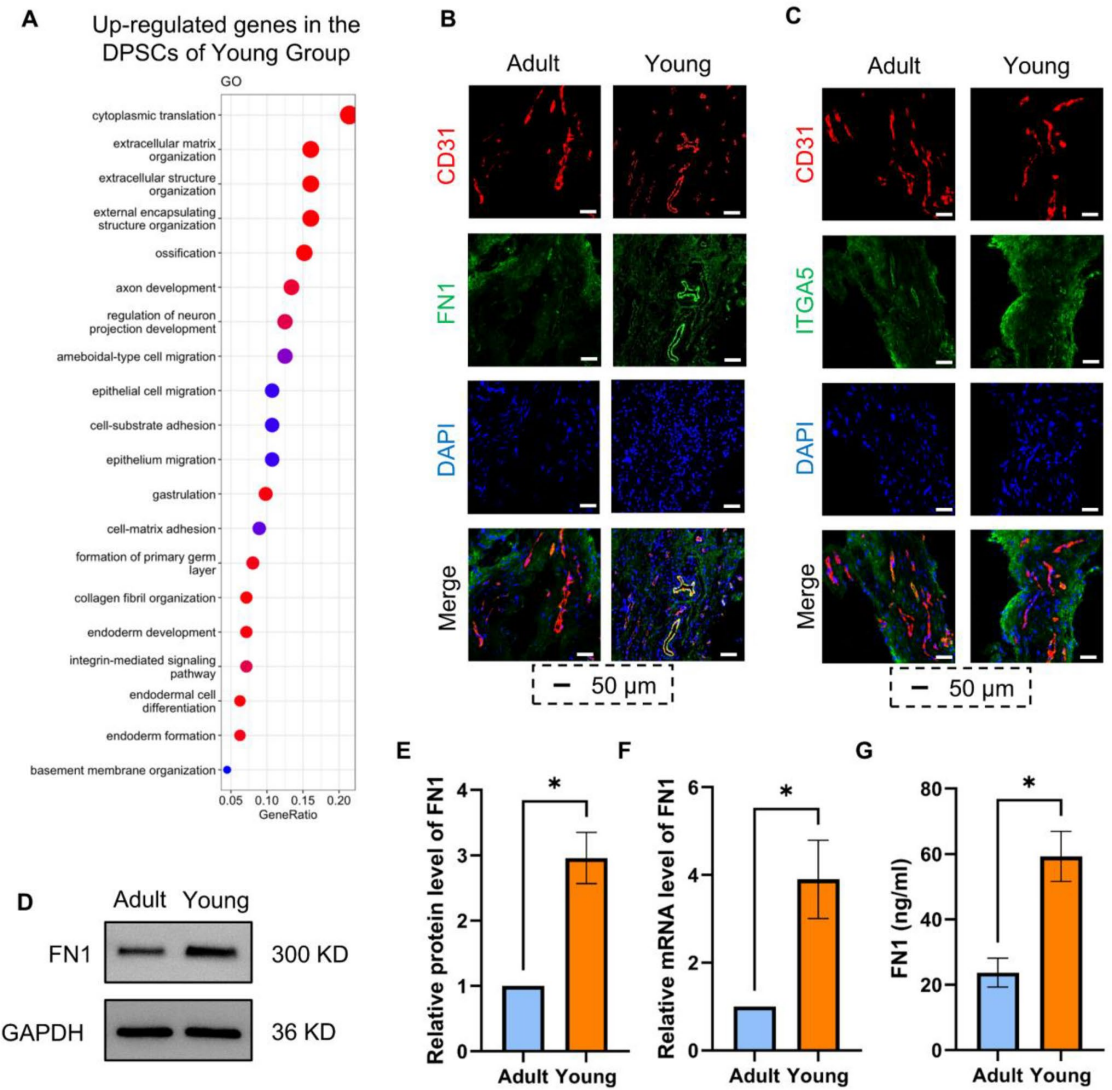
Differentially expression of FN1/ITGA5 in dental pulp tissue. **A**: GO analysis of gene expression in DPSCs from mature and young permanent dental pulp tissues. **B**: Immunofluorescence staining of CD31 (red) and ITGA5 (green) in mature and young permanent dental pulp tissues. **C**: Immunofluorescence staining of CD31 (red) and FN1 (green) in mature and young permanent dental pulp tissues. D–G: Western blot, RT-PCR, and ELISA were used to detect FN1 expression in A-DPSCs and Y-DPSCs (*P < 0.05, **P < 0.01, ***P < 0.001)

The differences between A-DPSCs and Y-DPSCs may have resulted in different effects on ECs. GO analysis results indicated that highly expressed genes in Y-DPSCs were primarily enriched in cytoplasmic translation and extracellular matrix structure (Fig. 4A), indirectly verifying the key role of FN1 in Y-DPSCs. We further studied the spatial expression of FN1/ITGA5 in dental pulp tissue to validate the specific roles of the FN1-ITGA5 signaling pathway in DPSC-EC intercellular communication. Immunohistochemistry results revealed co-expression of FN1 and CD31 in ECs in young and mature dental pulp tissues (Fig. 4B). High ITGA5 expression was observed around CD31 + blood vessels (Fig. 4C). Figure 2A also presents the location of DPSCs.

**Fig. 5:**
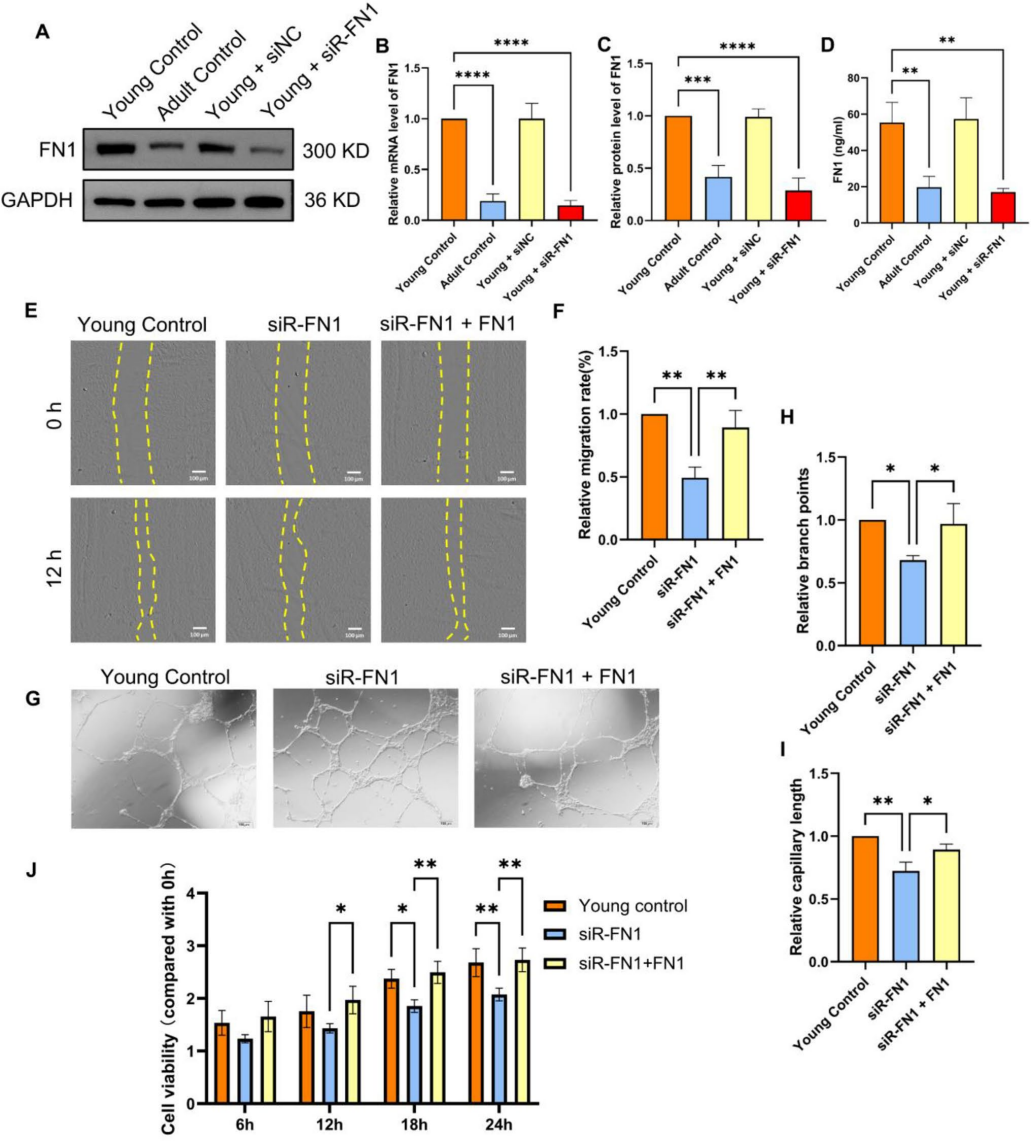
Regulatory effects of FN1 in DPSCs on the biological functions of co-cultured HUVECs. **A**–**D**: After silencing FN1 in Y-DPSCs, western blot, RT-PCR, and ELISA were detected to validate FN1 expression; **E**–**F**: Cell migration of HUVECs co-cultured with DPSCs in a scratch assay. **G**–**I**: The tube formation assay was performed by counting branch points and capillary length. **J**: Proliferative ability was determined using CCK-8 assay (*P < 0.05, **P < 0.01, ***P < 0.001, ****P < 0.0001)

Meanwhile, we extracted primary DPSCs from young and mature dental pulps and cultured them in vitro to further verify the FN1 expression in A-DPSCs and Y-DPSCs. ELISA, RT-qPCR, and Western blot were used to detect FN1 expression. Y-DPSCs and their conditioned medium expressed FN1 at significantly higher levels than A-DPSCs (Figs. 5D–G). These results supported the existence of the FN1-ITGA5 signaling pathway in DPSC-EC intercellular communication, warranting further study (Fig. 4).

### Regulatory effects of FN1 in DPSCs on the biological functions of co-cultured HUVECs

We investigated the influence of FN1 in DPSCs on the biological functions of co-cultured HUVECs via in vitro experiments. We performed FN1 siRNA transfection in Y-DPSCs and verified FN1 expression using Western blot, RT-PCR, and ELISA (Figs. 5A–D). Thus, we simulated a co-culture using a conditioned medium and compared the tube formation and wound-healing abilities of HUVECs. Scratch, tube formation experiments and CCK8 assays of HUVECs displayed that FN1 knockdown in Y-DPSCs significantly reduced the ability of HUVECs to heal wounds and form tubes while adding the corresponding FN1 to their medium restored the wound healing, tube formation and proliferation abilities of HUVECs compared to the Y-DPSCs control group (Figs. 5E–J). These results indicate that FN1 in DPSCs promotes the proliferation and tube formation capabilities of HUVECs (Fig. 5).

### FN1 regulates the tube formation function of HUVECs via ITGA5

**Fig. 6:**
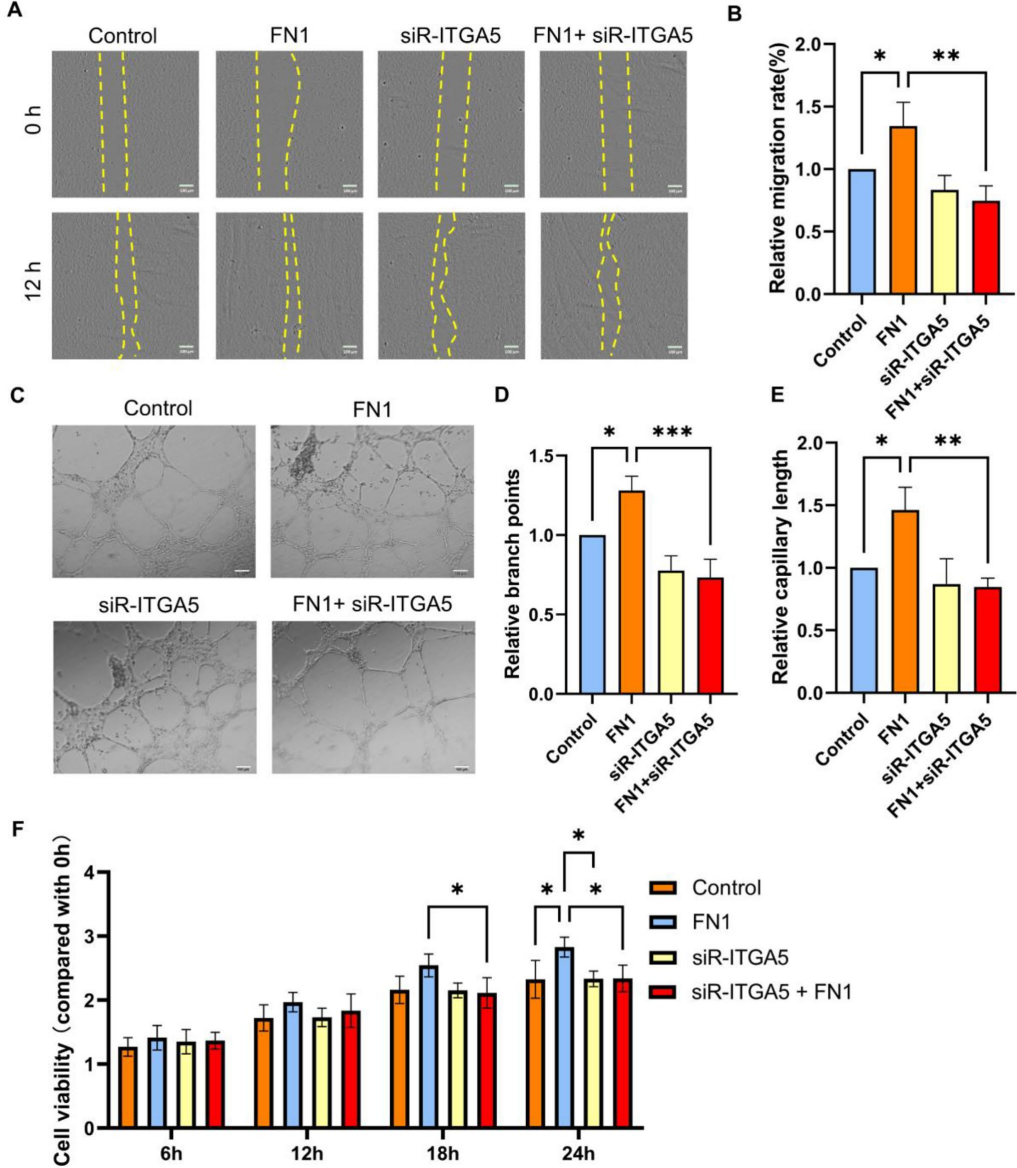
FN1 regulates the tube formation function of HUVECs via ITGA5. **A**/**B**: Cell migration of HUVECs treatment with FN1 or siR-ITGA5 in scratch assay. **C**–**E**: The tube formation assay of HUVECs treatment with FN1 or siR-ITGA5 was performed by counting branch points and capillary length. **F**: Proliferative ability was determined using CCK-8 assay (*P < 0.05, **P < 0.01, ***P < 0.001)

Based on previous experimental results, we hypothesized that FN1 in DPSCs regulates the tube formation function of HUVECs via ITGA5 and verified this via in vitro experiments. We divided HUVECs into the following four groups: Control group (no treatment), the FN1 culture group (FN1 of 50 ng/mL), the ITGA5 knockdown group, and the ITGA5 knockdown + FN1 culture group. Scratch (Fig. 6A/B), tube formation (Figs. 6C–E) experiment and and CCK8 assays of HUVECs(Fig. 6F) indicated that HUVECs cultured with FN1 in the medium had significantly enhanced healing rate, tube formation and proliferation ability, while FN1 had little effect on ITGA5 knockdown HUVECs compared to the Control group. These results indicate that FN1 primarily promotes the healing and tube formation abilities of HUVECs via ITGA5 (Fig. 6).

### Animal experiments demonstrated that silencing FN1 or ITGA5 in dental pulp tissue exerts inhibitory effects on dental pulp vascular development

**Fig. 7:**
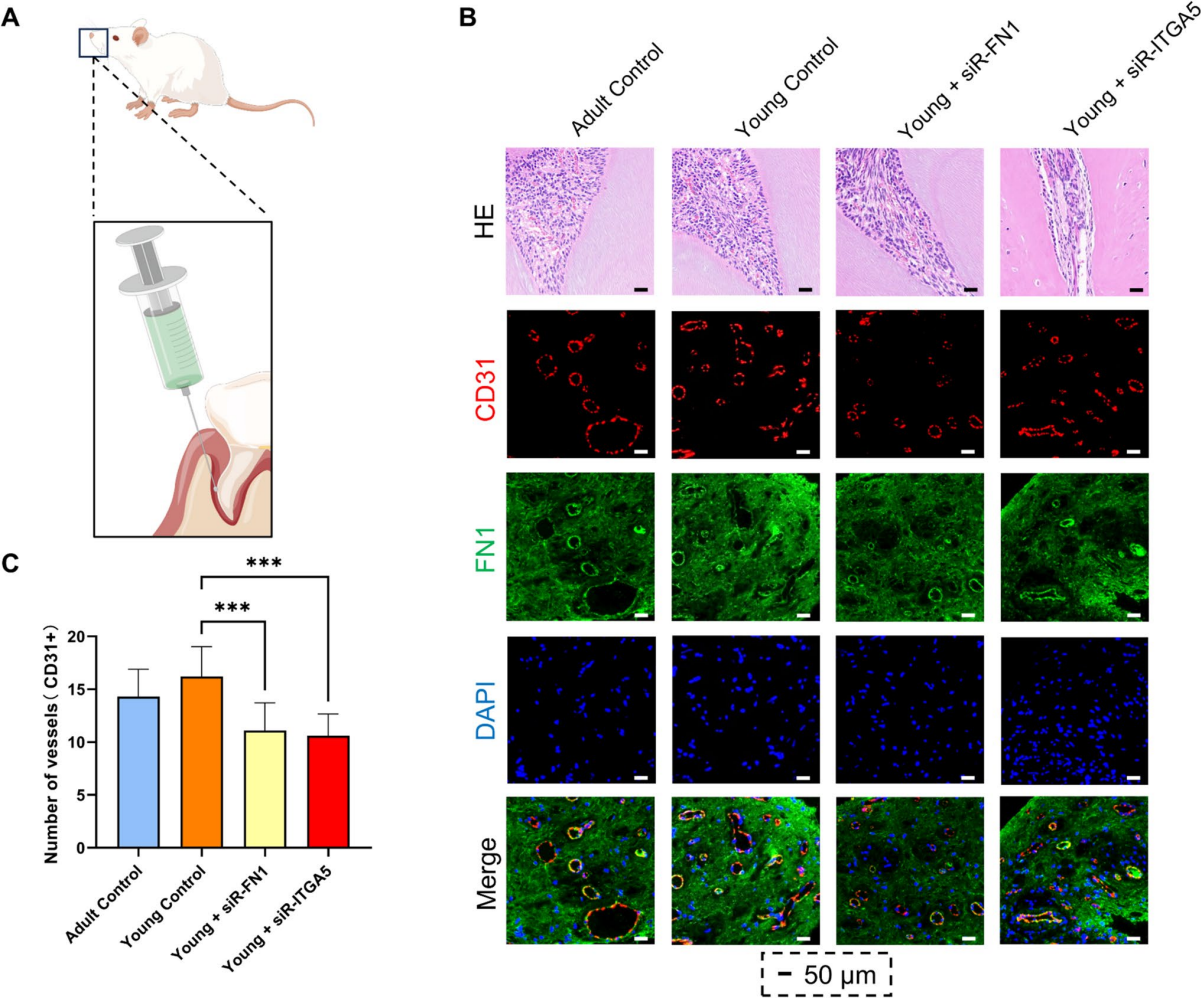
Silencing FN1 or ITGA5 in dental pulp tissue exerts inhibitory effects on dental pulp vascular development. **A**: Inhibitory modulation of FN1 and ITGA5 functionality in developing dental pulp tissue was achieved through periodontal ligament injections of siRNA targeting FN1 or ITGA5, respectively, in mice. **B**: Immunofluorescence staining of CD31 (red) and FN1 or ITGA5 (green) in mature and young permanent dental pulp tissues. **C**: Numbers of vessels in dental pulp tissues of each group (*P < 0.05, **P < 0.01, ***P < 0.001)

We employed periodontal ligament injections of siRNA to suppress the expression of FN1 or ITGA5 in mouse dental pulp tissue (Figs. 7A). Results revealed a significant reduction in FN1 expression in young dental pulp tissue upon siRNA treatment (Figs. 7B). Partial inhibition of FN1 or ITGA5 in mouse dental pulp tissue led to a noticeable suppression of dental pulp vascular development (Figs. 7C). This outcome, derived from animal experiments, further substantiates our earlier research conclusion that FN1 and ITGA5 play pivotal roles in the process of dental pulp vascular development (Fig. 7).

### FN1/ITGA5 regulates the tube formation function of HUVECs via the PI3K/AKT signaling pathway

Previously, we discovered that the FN1/ITGA5 axis in intercellular communication can regulate the tube formation function of HUVECs. We speculated that the downstream PI3K/AKT signaling pathway may play a key role in this process based on the KEGG and GO analysis results of the transcriptional profiles of ECs in dental pulp tissue from single-cell sequencing.

**Fig. 8:**
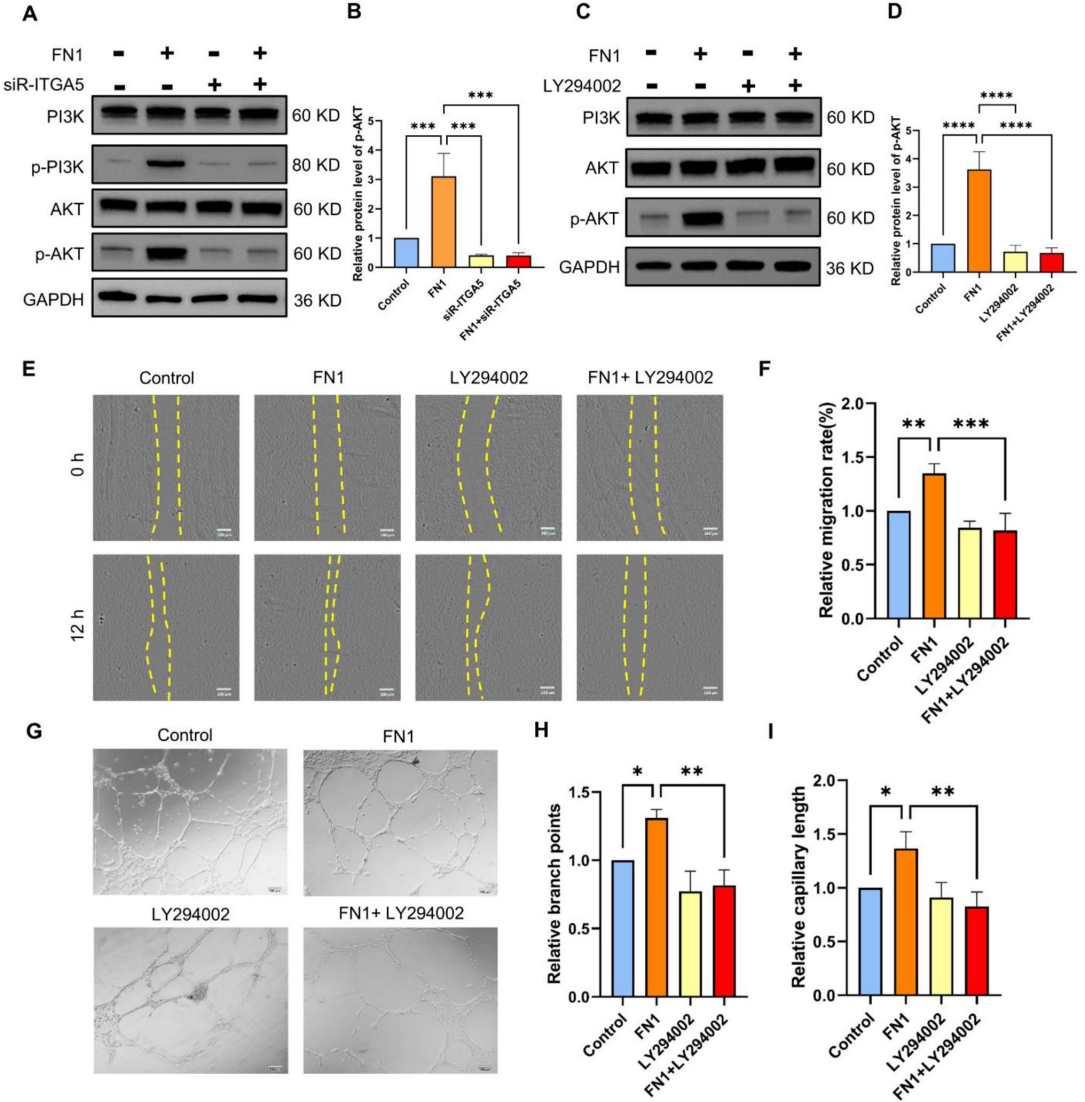
FN1/ITGA5 regulates the tube formation function of HUVECs via the PI3K/AKT signaling pathway. **A**/**B**: Evaluation of PI3K/AKT signaling pathway activation status in HUVECs treated with FN1 or siRNA using Western blot. **C**/**D**: Evaluation of PI3K/AKT signaling pathway activation status in HUVECs treated with FN1 or LY294002 using Western blot. **E**/**F**: Cell migration of HUVECs treatment with FN1 or LY294002 in scratch assay. **G**–**I**: The tube formation assay of HUVECs treatment with FN1 or LY294002 was performed by counting branch points and capillary length (*P < 0.05, **P < 0.01, ***P < 0.001, ****P < 0.0001)

HUVECs were divided into the following four groups: Control group (no treatment), FN1 culture group (FN1 of 50 ng/mL), ITGA5 knockdown group, and ITGA5 knockdown + FN1 culture group. The PI3K/AKT signaling pathway activation was detected in each group using Western blot. Figure 8A/B presents that the hosphor-PI3K and hosphor-AKT levels in HUVECs increased significantly after adding FN1 to the culture medium but did not change significantly after treating ITGA5 knockdown HUVECs with FN1 compared to the Control group.

Furthermore, we used LY294002 to inhibit the PI3K/AKT signaling pathway to examine its role in the FN1/ITGA5 axis, regulating HUVECs’ wound healing and tube formation abilities (Fig. 8C/D). Scratch (Fig. 8E/F) and tube formation (Figs. 8G–I) experiment results showed that HUVECs cultured with FN1 in the medium had significantly enhanced healing and tube formation abilities, while inhibition of the PI3K/AKT signaling pathway significantly reduced the effect of FN1 on promoting HUVECs’ tube formation ability compared to the Control group. These results indicate that the FN1/ITGA5 axis can activate the PI3K/AKT signaling pathway to further promote HUVECs’ wound healing and tube formation abilities (Fig. 8).

## Discussion

Tooth loss and pulp necrosis caused by various reasons seriously affect children's daily lives and growth and development. However, the success rate of current pulp regeneration therapy remains low due to the lack of an effective method to address this problem [1]. Exploring the regulatory mechanisms of pulp development is fundamental to achieving pulp regeneration. This study analyzed the gene expression of DPSCs and ECs in developing and mature pulp tissues and their intercellular communication with ECs by mining public databases and performing single-cell sequencing. It explored and demonstrated the important role of FN1/ITGA5-mediated DPSC-EC intercellular communication in vascular generation during pulp development.

Tooth health is closely related to basic physiological functions such as eating and speaking. However, acute trauma or chronic stimulation frequently damages the teeth, resulting in tooth loss and pulp removal, seriously affecting daily life. Regenerative medicine holds promise for the physiological repair of damaged teeth. For example, pulp regeneration therapy is a current research hotspot [3, 4, 22]. Dental stem cells, such as DPSCs, stem cells from apical papilla (SCAP), and SHEDs, play a key role in pulp regeneration [3, 13, 24–28]. DPSCs have multidirectional differentiation potential, such as vascular differentiation [29, 30]. They can be easily obtained from third molars and orthodontically extracted teeth, making them an excellent cell source for pulp regeneration [31, 32]. The primary research approaches include transplanting exogenous stem cells and homing autologous stem cells to achieve pulp regeneration [3, 4, 22]. Regardless of approach, promoting pulp vascularization in the early stage of treatment is crucial to improve success rate.

Neovascularization is crucial in physiological processes, such as growth, development, and wound healing [33]. New blood vessels can deliver nutrients and maintain microenvironment stability, necessary for cell proliferation and tissue regeneration [34]. Vascularization involves local activation of ECs. Activated ECs in granulation tissue interact with ECM to form new capillaries in the wound-healing microenvironment via proliferation and migration [35]. This study compared the gene expression differences of ECs between developing and mature pulp tissues using single-cell sequencing. We discovered significant differences in many key genes related to cell proliferation and migration. Understanding the complex roles and applications of stem cells in vascularization is a current difficulty, as vascularization involves complex direct and indirect signaling regulation networks [36]. Studying regulatory mechanisms during development to guide pulp regeneration is a currently focused research approach [37]. Since DPSCs are frequently located around blood vessels, this indicates a possible reciprocal regulation between DPSCs and ECs [22]. This study demonstrated in vitro and in vivo that perivascular DPSCs can promote the proliferation, migration, and tube formation of ECs, and it explored their potential regulatory mechanisms.

Intercellular communication analysis was used to study the interaction mechanisms between DPSCs and ECs in pulp tissue during development. ECs require regulatory effects from adjacent cells for activation because the primary cell type is involved in neovascularization. In this process, ECs receive regulation from the hypoxic microenvironment and interact with neighboring cells to migrate within fibrin/fibronectin-rich clots. ECs respond to pro-angiogenic signals, such as VEGF, FGF, PDGF-B, TGF-β, and vascular endothelial growth factor signaling pathways, to proliferate and migrate, thereby initiating vascularization [38]. Intercellular communication analysis presented that FN1-ITGA5-mediated DPSC-EC intercellular communication was more active in developing pulp tissue. While FN1 has been extensively studied in the context of embryonic development in humans and animals, limited research has been conducted on its role in dental pulp tissue development [39–42]. Specifically, there are few articles about the specific involvement of FN1 in the dental developmental process.

Considering the importance of EC-DPSCs interactions in vascularization, in vitro experiments verified that DPSCs can promote proliferation, migration, and tube formation of ECs via FN1-ITGA5-mediated intercellular communication. Integrin receptors on ECs, especially ITGA5, have previously been widely studied in vascularization [38]. This study identified and validated that FN1-ITGA5-mediated intercellular communication during pulp development can promote the proliferation, migration, and tube formation of ECs. Based on this, we verified that the PI3K/AKT signaling pathway downstream of ITGA5 plays an important role using gene expression analyses in young pulp ECs from single-cell sequencing data. The PI3K/AKT signaling pathway is important in promoting vascularization, inhibiting EC inflammation and apoptosis [43–47], and can be regulated by integrin-related signals, including ITGA5 [48–52]. This conclusion indicates that the downstream PI3K/AKT signaling pathway of FN1-ITGA5-mediated intercellular communication also has potential as a regulatory target in vascular-related research.

However, this study has certain limitations. For example, transwell chambers were primarily used for co-culture experiments in vitro, failing to fully simulate the spatial relationship between DPSCs and ECs during pulp growth and development in vivo. We may use direct contact co-culture in vitro and animal experiments in the future to further validate this conclusion. Additionally, how to apply this study’s findings to promote pulp neovascularization remains controversial, and we will study gene editing or screening of DPSCs based on the current research results to facilitate clinical application in the next step.

## Summary

This study comprehensively characterized the gene expression of DPSCs and ECs and their intercellular communication with vascular ECs in young and mature pulp tissues by mining public databases and performing single-cell sequencing analysis. We compared the regulatory effects between DPSCs and ECs in young and mature pulp tissues. We identified and validated that FN1-ITGA5-mediated DPSC-EC intercellular communication and its downstream PI3K/AKT signaling pathway are important promoters of neovascularization in developing pulp tissue. We aim to underscore the novelty and importance of our findings in the broader context of biological development. Through single-cell sequencing, cellular experiments, and animal studies, we have demonstrated the significance of FN1 in the dental pulp vascular development. Notably, we have identified DPSCs as a crucial source of FN1 in dental pulp tissues. This novel finding lays the foundation for our further exploration of how FN1 + DPSCs can promote angiogenesis. In future research, we aim to delve into the specific mechanisms underlying the pro-angiogenic effects of FN1 + DPSCs, with the ultimate goal of advancing dental pulp regeneration therapies and improving wound healing.

### Supplementary Information

Below is the link to the electronic supplementary material.

Supplementary file1 (CSV 63 KB)

Supplementary file2 (CSV 70 KB)

Supplementary file3 (CSV 53 KB)

Supplementary file4 (CSV 8 KB)

Supplementary file5 (CSV 38 KB)

Supplementary file6 (CSV 3 KB)

Supplementary file7 (CSV 155 KB)

Supplementary file8 (CSV 13 KB)

Supplementary file9 (CSV 45 KB)

Supplementary file10 (CSV 2 KB)

Supplementary file11 (TIF 2.79 KB)

## Data Availability

The datasets and materials used or analysed during the current study are available from the corresponding author on reasonable request.
