# Supplementary figures and images for: Enhancing Vasculogenesis in Dental Pulp Development: DPSCs-ECs Communication via FN1-ITGA5 Signaling
Source: Stem Cell Rev Rep. 2024 Feb 28;20(4):1060–77. doi: 10.1007/s12015-024-10695-6 (PMC11087358; doi:10.1007/s12015-024-10695-6)

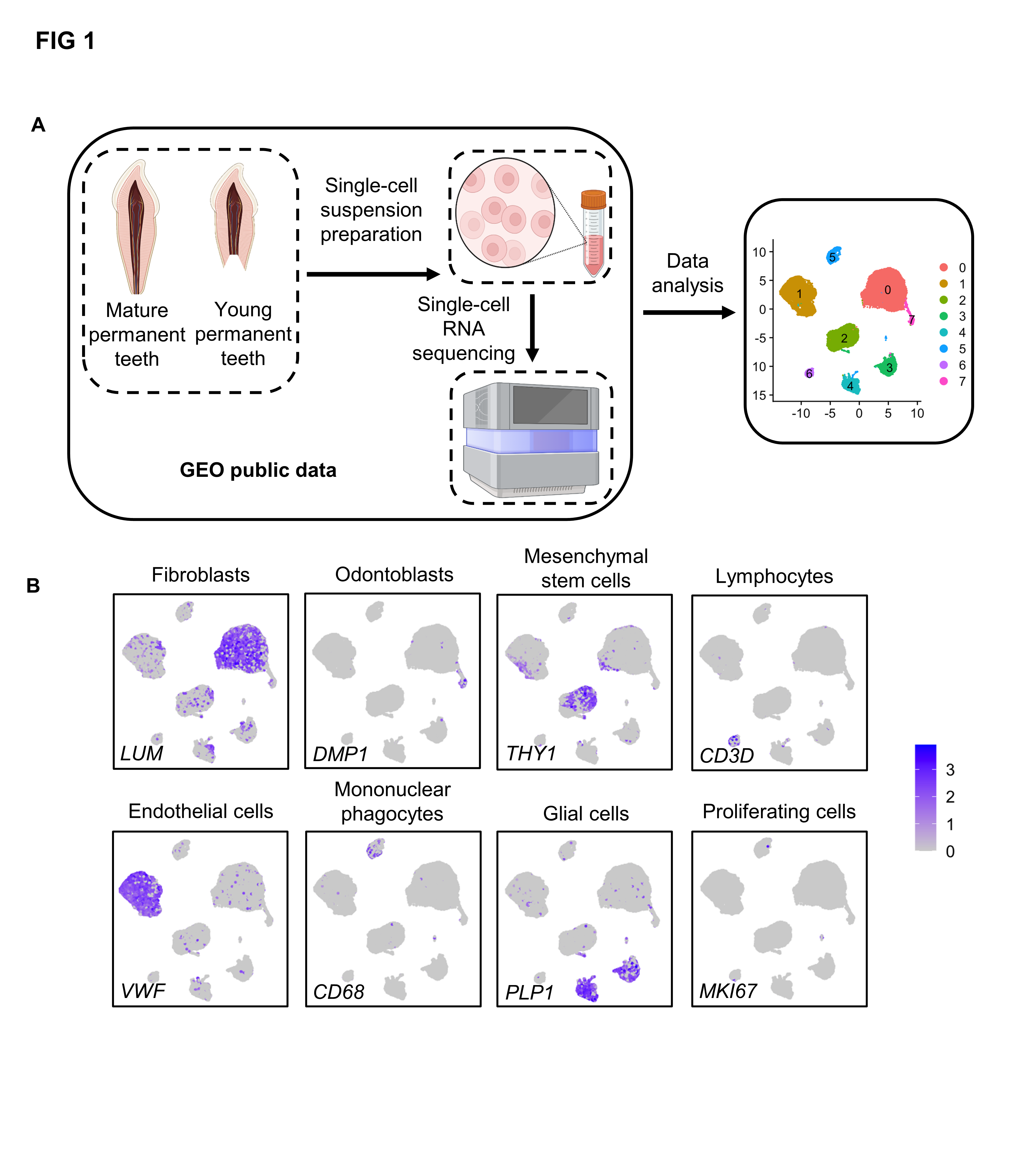

Supplement: Supplementary file 11 — Supplementary file11 (TIF 2.79 KB) [file 12015_2024_10695_MOESM11_ESM.tif]
